# Adaptive Observer Based Fault Tolerant Control for Sensor and Actuator Faults in Wind Turbines

**DOI:** 10.3390/s21248170

**Published:** 2021-12-07

**Authors:** Jing Teng, Changling Li, Yizhan Feng, Taoran Yang, Rong Zhou, Quan Z. Sheng

**Affiliations:** 1 School of Control and Computer Engineering, North China Electric Power University, Beijing 102206, China; jing.teng@ncepu.edu.cn (J.T.); lichangling223@163.com (C.L.); yizhan.feng@ncepu.edu.cn (Y.F.); ytr774082659@163.com (T.Y.); zhourong@ncepu.edu.cn (R.Z.); 2 School of Computing, Macquaire University, Sydney, NSW 2109, Australia

**Keywords:** fault tolerant control, adaptive observer, fast adaptive fault estimation (FAFE) algorithm, wind turbines

## Abstract

The installed wind energy generation capacity has been increasing dramatically all over the world. However, most wind turbines are installed in hostile environments, where regular operation needs to be ensured by effective fault tolerant control methods. An adaptive observer-based fault tolerant control scheme is proposed in this article to address the sensor and actuator faults that usually occur on the core subsystems of wind turbines. The fast adaptive fault estimation (FAFE) algorithm is adopted in the adaptive observers to accurately and rapidly located the faults. Based on the states and faults estimated by the adaptive observers, the state feedback fault tolerant controllers are designed to stabilize the system and compensate for the faults. The gain matrices of the controllers are calculated by the pole placement method. Simulation results illustrate that the proposed fault tolerant control scheme with the FAFE algorithm stabilizes the faulty system effectively and performs better than the baseline on the benchmark model of wind turbines.

## 1. Introduction

Wind energy contributes to a large part of power production worldwide as a type of clean and renewable energy. With the expansion of the scale of wind turbines, their maintenance costs are correspondingly surging. To reduce maintenance costs, enhance reliability, and prolong the life expectancy of wind turbines, researchers have focused on effective fault tolerant control (FTC) techniques.

The two principal FTC techniques are the passive fault-tolerant control (PFTC) and the active fault-tolerant control (AFTC). The PFTC methods design the controller based on the knowledge of the possible fault sets of the system, whereas the AFTC ones rely on the online information provided by the fault estimation or detection and isolation techniques. These approaches adopt distinctive design methodologies, resulting in different properties. Specifically, the PFTC methods are robust against a specified class of faults or uncertainties of the whole system [1,2,3]. However, the stability of the system is the first consideration of the PFTC methods, making them more conservative from the performance viewpoint, thus making it challenging to achieve optimal performance [4,5]. Furthermore, their fault tolerant capabilities are limited since fault detection schemes are not compulsory in the PFTC approaches. In contrast, the AFTC ones rely on the real-time information provided by fault estimation or fault detection and isolation techniques. Accordingly, the control system can be reconfigured to actively accommodate the faults, thus ensuring the stability of the system. The AFTC methods have been widely applied to various systems, including the Takagi-Sugeno (TS) fuzzy systems, stochastic systems, singularly perturbed systems (SPS), nonlinear systems, and interconnected systems. Specifically, in the TS fuzzy systems, an adaptive observer was adopted in [6] to estimate the time-varying faults, and the corresponding controller was designed. Considering the Brownian parameter perturbations, process uncertainties, and faults in the TS fuzzy systems, Liu et al. constructed an unknown input observer with sliding mode terms [7]. The authors then proposed using the feedback gain and the sensor compensation output to stabilize the state and eliminate the influence of faults. To deal with the stochastic systems with the faults and disturbances, Sun et al. in [8] presented an anti-disturbance FTC solution based on the estimated information obtained by the stochastic disturbance observer and the fault diagnosis observer. A global fault-tolerant controller was proposed for the time-delayed singularly perturbed systems (SPS) in [9]. The global controller consisted of the Luenberger observer-based controller and the state feedback controller, which were separately designed for the fast and slow subsystems. Regarding the nonlinear systems, Li et al. developed a fuzzy fault tolerant optimal control method based on the adaptive backstepping technique in [10] to solve the unknown actuator faults. Considering the interconnected systems, Yang et al. [11] implemented a fault tolerant control scheme, which applied the fault diagnostic observer and combined both the unconstrained and constrained interconnected separation principle. The AFTC approaches are more flexible than the PFTC ones in dealing with faults. Besides, the AFTC methods can achieve the best possible performance [5], given accurate and timely diagnostic information and sufficient reaction time for controller synthesis. The AFTC scheme is adopted to stabilize the faulty wind turbine system in our study because of these advantages.

A vital step of the AFTC scheme is to online estimate the fault for the fault tolerant controller. Liu et al. designed an unknown input observer in [12], where the fault signal and the modeling uncertainty were combined as the unknown input of the observer. However, the coupling between the state estimation error and the disturbance should be addressed by H${}_{\infty }$ optimization, which increased the complexity of the design. More convenient methods to estimate the faults can be found in references [13,14]. They augmented the faulty system to directly estimate the fault signal with limited estimation accuracy. In contrast, the adaptive observer could estimate the system state and fault values simultaneously. Moreover, the fault estimation accuracy could be improved by adjusting the adaptive learning rate or applying adaptive algorithms. Therefore, the adaptive observer has drawn researchers’ attention. For instance, the adaptive observers were selected as fault estimation units in [15] for the pitch system and in [16] for the linear system. However, calculating the correlation matrices of fault estimation is challenging given the linear matrix inequality (LMI). The fast adaptive fault estimation (FAFE) algorithm proposed in [17,18] solved the correlation matrices given the LMI conditions while reducing the complexity of the observer design. Nevertheless, the FAFE algorithm has not been applied in the wind turbine system to the authors’ knowledge. The conventional fault estimation algorithm was utilized for the wind turbine system in [19,20], where the former focused on the pitch and converter subsystems, and the latter considered the winding short circuit fault. However, the conventional algorithm is not suitable for the time-varying faults, whose fault estimation performance is unsatisfactory. The FAFE algorithm mentioned above can trace the actual value faster and have higher estimation accuracy than the conventional one for both the constant and time-varying faults. Therefore, the FAFE algorithm is adopted in this research to design the adaptive observers of wind turbines.

To propose an effective AFTC scheme, we should analyze the wind turbine state at the system level. The benchmark model proposed in [21,22] made some simplifications and assumptions of the wind turbines to provide a system-level model for studying fault diagnosis and fault-tolerant control methods with acceptable performance. Therefore, the benchmark model has been widely applied for wind turbine analysis. This model comprises four subsystems: the blade and pitch subsystem, drive train, generator and converter, and controller. The system faults, actuator faults, and sensor faults are considered in the benchmark model during the operating time. Based on this model, scholars have presented various solutions to realize fault detection [23,24] and fault tolerance, thus increasing the stability of wind turbines. For instance, the reference [23] adopted the Gibbs sampling algorithm for sensor fault detection, and the Fuzzy/Bayesian network was utilized to classify the fault type for fault isolation. In [24], the fault diagnosis and isolation scheme for sensor and system faults was accomplished by combining the negative selection algorithm and the smoothing moving window filter. As for the fault tolerance scheme, an adaptive discrete-time linear controller was proposed in [25] for the benchmark model, which was designed based on the online identification scheme. Feng et al. utilized both analytical and hardware redundancies to realize the AFTC for sensor faults [26]. The healthy redundant sensor provided estimated system state information when the sensor fault was detected. However, the introduction of redundant sensors increased the cost. The adaptive observer based AFTC method presented in this paper needs no hardware redundancy scheme, thus reducing the cost. A PI controller method is designed for the benchmark model based on the data-driven residual generator in [27], and an optimal scheme was introduced to make the generator sensitive to the considered faults. An output feedback control scheme based on the extended state observer was presented for the sensor and actuator faults [28]. In this literature, the augmented system state vector contained the considered faults and the system state. A similar method to construct the augmented system can be referred to [29]. The unknown input and disturbance simultaneously influence the drive train subsystem. Thus the authors designed an unknown input observer and a state feedback controller to realize the AFTC objective. However, the fault estimation accuracy of the augmented system was limited. In contrast, the adaptive observer with the FAFE algorithm adopted in our study obtained a considerable fault estimation performance. To deal with the actuator fault in the blade and pitch subsystem, the authors introduced the Proportional-Integral (PI) controller [30]. The adaptive step-by-step sliding mode observer provided the estimated state and fault information. This work was extended in [31], in which the robustness of different individual pitch control (IPC) systems was verified. However, the PI controller chosen in these research works may decrease the stability of the system. To address the actuator faults, Jain et al. developed the fault detection and controller reconfiguration modules in [32], which adopted the unknown input residual generator and the model-predictive control technique. The FTC scheme for the sensor fault in the wind turbine system under the partial load condition was presented by Ghanbarpour et al. in [33], where the sliding mode observer estimated the faults and the predictive model controller achieved maximum power extraction. Based on the current research results, the authors considered the sensor and actuator faults simultaneously and proposed a PFTC scheme in their recent work [34].

Based on the benchmark model, we designed an adaptive observer for fault detection in wind turbines in [35]. The adaptive observer calculated the estimated value and the residuals to detect the faults. Based on the adaptive observer proposed in [35], we further realized the AFTC, thus the performance of wind turbines in faulty conditions could be stabilized. Our simulation is based on the benchmark model representing a three-bladed pitch-controlled variable-speed wind turbine with a nominal power of 4.8 MW. Both the sensor and actuator fault scenarios occur on the blade and pitch subsystem and the generator and converter subsystem which are simultaneously considered in this study. To address these faults, we reconstruct the faulty system as the linear form. The linear observation technique is a direct method that simplifies the motion equation of the dynamic system. It results in a relatively low calculation cost, fast response speed, and a compact system matrix for controlling wind turbines. The adaptive observer and the FAFE algorithm are suitable for estimating the unknown parameters in the linear system and with better performance. The estimated values can be further used in the state feedback fault tolerant controller. The main contributions of this paper are as follows: (1) The two subsystem models are reconstructed as faulty forms according to the sensors’ measured values. Based on these models, we designed corresponding adaptive observers to estimate the system states and faults. (2) We applied the FAFE algorithm in the adaptive observers to enhance the fault estimation performance. (3) According to the estimated information from the adaptive observers, the state feedback controllers are designed to realize adaptive fault tolerant control, where the pole placement method is adopted to calculate the gain matrices.

Simulation results illustrated that the proposed method effectively realized fault tolerant control for the considered faults in wind turbines. Furthermore, we also compared the fault estimation and fault tolerant control performance by adopting the FAFE algorithm with the contrast experiment in designing the adaptive observer. The results proved that the FAFE algorithm performed much better than the baseline.

The remainder of this paper is organized as follows. Section 2 briefly reviews the benchmark model. In Section 3, the adaptive observer adopting the FAFE algorithm is presented, and Section 4 proposes the state feedback controller. Section 5 shows the simulation results and proves the effectiveness of the proposed FTC method. The conclusions are drawn in Section 6.

## 2. The Wind Turbine Benchmark Model

**Figure 1:**
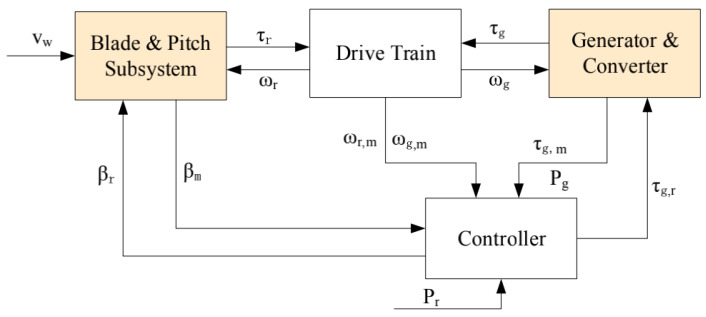
The wind turbine benchmark model.

The wind turbine benchmark model proposed in [22] is a three-blade turbine with a full converter coupling, which comprises four parts: the blade and pitch subsystem, the drive train, the generator and converter, and the controller, as illustrated in Figure 1. It is a variable-speed and pitch-controlled turbine. The variables between the four subsystems are defined as follows: the wind speed denoted by $v_{w}$ acts on the blades and goes into the blade and pitch subsystem. The controller provides the pitch position reference $\beta_{r}$ to the blade and pitch subsystem and the torque reference $\tau_{g,r}$ to the generator. $\beta_{m}$ is the measured pitch position. The drive train converts the rotor torque $\tau_{r}$ and the generator torque $\tau_{g}$ to the rotor speed $\omega_{r}$ and the generator speed $\omega_{g}$. The rotor speed and the generator speed are separately measured by $\omega_{r,m}$ and $\omega_{g,m}$. The generator and converter subsystem utilizes the generator reference torque $\tau_{g,r}$ to obtain the torque $\tau_{g}$. $P_{g}$ is the power produced by the generator, which follows the reference power $P_{r}$ through the controller. The blade and pitch subsystem and the generator and converter subsystem are the critical parts of wind turbines, denoted by the orange rectangles in Figure 1. We research the two subsystems and their corresponding fault scenarios in detail in this study, the relevant parameters in these two subsystems are consistent with those in the reference [22].

### 2.1. The Blade and Pitch Subsystem Model

The aerodynamic and the pitch models constitute the blade and pitch subsystem. The aerodynamic is modeled as the torque acting on the blade. The hydraulic pitch system is a piston servo system, which can be modeled by a second order transfer function between the *i* th pitch angle $\beta_{i}$ and the corresponding reference $\beta_{i,r}$ [22]:

(1) $$\frac{\beta_{i}\left(s\right)}{\beta_{i,r}\left(s\right)}=\frac{\omega_{n}^{2}}{s^{2}+2\cdot \zeta \omega_{n}\cdot s+\omega_{n}^{2}}.$$

We transform the system from the time domain into the complex frequency domain, where s=σ+jω represents the complex plane, ζ=0.7 is the damping factor, and $\omega_{n}=11.11$ is the natural frequency.

### 2.2. The Generator and Converter Model

The electrical system in the wind turbine and the electrical system controllers are much faster than the frequency range used in the benchmark model. On a system level of the wind turbine, the generator and converter subsystem is described as a first-order transfer function between the generator torque $\tau_{g}$ and the reference torque $\tau_{g,r}$:

(2) $$\frac{\tau_{g}\left(s\right)}{\tau_{g,r}\left(s\right)}=\frac{\alpha_{gc}}{s+\alpha_{gc}},$$

where $\alpha_{gc}=50$ denotes the generator & converter model parameter.

The power $P_{g}$ produced by the generator is calculated by:

(3) $$P_{g}=\eta_{g}\omega_{g}\tau_{g},$$

where $\eta_{g}=0.98$ [22] denotes the generator efficiency, $\omega_{g}$ is the generator speed, and $\tau_{g}$ is the generator torque.

### 2.3. Fault Scenarios

All the described faults originate from the actual faults in wind turbines. The sensor and the actuator faults mainly occur in the blade and pitch subsystem and the generator and converter subsystem. The corresponding fault scenarios are summarized in Table 1. As can be seen from the table, the sensor faults have two manifestations: the fixed value and the gain factor. The actuator faults may change the dynamics of the pitch system while resulting in an offset on the converter. Concerning mathematical modeling, both additive and multiplicative faults as well as faults resulting in changing dynamics of parts of the system are considered in this benchmark model.

## 3. Adaptive Observer Design

Two adaptive observers are designed for the blade and pitch subsystem and the generator and converter subsystem. Both the two subsystems are remodeled to be linear. Owing to the linearization, the corresponding observers can successfully estimate both the system states and faults. Specifically, the fault estimation performance is enhanced by applying the FAFE algorithm. The estimated information is further utilized in the fault tolerant control procedure of Section 4.

### 3.1. Adaptive Observer for the Blade and Pitch Subsystem

According to the transfer function Equation (1) of the blade and pitch subsystem, we remodeled it by the linear state-space form:

(4) $$\begin{array}{cc} \; & \dot{x_{p}^{i}}=\left[\begin{array}{cc} -2\zeta \omega_{n} & -\omega_{n}^{2}\\ 1 & 0 \end{array}\right]x_{p}^{i}+\left[\begin{array}{c} 1\\ 0 \end{array}\right]\beta_{r}^{i},\\ \; & y_{p}^{i}=\left[\begin{array}{cc} 0 & \omega_{n}^{2} \end{array}\right]x_{p}^{i}, \end{array}$$

where $x_{p}^{i}=\left[\begin{array}{c} \dot{\beta_{i}}\\ \beta_{i} \end{array}\right]$ is the immeasurable state vector of the *i*-th pitch angle, $\dot{\beta_{i}}$ is the angular speed. $\beta_{r}^{i}$ is the input vector, and $y_{p}^{i}$ is the measurable output vector. To uniformly design the adaptive observer later, we denote the matrices as $\left[\begin{array}{cc} -2\zeta \omega_{n} & -\omega_{n}^{2}\\ 1 & 0 \end{array}\right]=A_{p}$, $\left[\begin{array}{c} 1\\ 0 \end{array}\right]=B_{p}$, $\left[\begin{array}{cc} 0 & \omega_{n}^{2} \end{array}\right]=C_{p}$. Therefore, the state-space model of the blade & pitch subsystem can be written as:

(5) $$\begin{array}{cc} \; & \dot{x_{p}^{i}}=A_{p}x_{p}^{i}+B_{p}\beta_{r}^{i},\\ \; & y_{p}^{i}=C_{p}x_{p}^{i}. \end{array}$$

the pair $\left(A_{p},B_{p}\right)$ is controllable and the pair $\left(A_{p},C_{p}\right)$ is observable.

Here, the fault types under consideration include the sensor and the actuator fault. Two sensors are utilized to measure all three pitch positions, ensuring physical redundancy of the measurements, denoted by $\beta_{i,m1}$ and $\beta_{i,m2}$, respectively. We define a variable $\Delta \beta_{i,m}$ to describe the pitch angle measurement error resulting from the sensor and the actuator faults:

(6) $$\Delta \beta_{i,m}=\beta_{i}-0.5\left(\beta_{i,m1}+\beta_{i,m2}\right).$$

when no fault occurs, we have $\beta_{i,m1}=\beta_{i,m2}=\beta_{i}$, which means $\Delta \beta_{i,m}=0$, standing for healthy system. Therefore, the purpose of fault diagnosis is to produce an accurate estimate of the faulty behavior $\Delta \beta_{i,m}$. Taking $\Delta \beta_{i,m}$ into consideration, we thus remodel the blade and pitch subsystem into the following form:

(7) $$\begin{array}{cc} \; & {\dot{x}}_{p}^{i}=A_{p}x_{p}^{i}+B_{p}\left(\beta_{r}^{i}+\Delta \beta_{i,m}\right),\\ \; & y_{p}^{i}=C_{p}x_{p}^{i}. \end{array}$$

To estimate $\Delta \beta_{i,m}$, the following adaptive observer is designed:

(8) $$\begin{array}{cc} \; & {\dot{\hat{x}}}_{p}^{i}=A_{p}{\hat{x}}_{p}^{i}+B_{p}\beta_{r}^{i}+B_{p}{\hat{f}}_{p}-L_{p}\left({\hat{y}}_{p}^{i}-y_{p}^{i}\right),\\ & {\hat{y}}_{p}^{i}=C_{p}{\hat{x}}_{p}^{i}, \end{array}$$

where ${\dot{\hat{x}}}_{p}^{i}$ is the estimated value of the system state ${\dot{x}}_{p}^{i}$, and ${\hat{y}}_{p}^{i}$ is the estimated output $y_{p}^{i}$. $L_{p}$ is the observer gain matrix, and ${\hat{f}}_{p}$ denotes the fault value to be estimated.

### 3.2. Adaptive Observer for the Generator and Converter Subsystem

According to the transfer function of the Generator and Converter subsystem defined in Equation (2), we define the state-space model of this linear subsystem as follows:

(9) $$\begin{array}{cc} \; & {\dot{x}}_{g}=-\alpha_{gc}x_{g}+\alpha_{gc}\tau_{g,r},\\ & y_{g}=x_{g}, \end{array}$$

where $x_{g}=\tau_{g}$ denotes the immeasurable state vector, and $y_{g}$ is the observation of the generator torque.

Unlike the redundant sensor configuration in the Blade and Pitch subsystem, only one sensor is used to measure the generator torque in the Generator and Converter subsystem. We define the measured value of the generator torque $\tau_{g,m}$ as:

(10) $$\tau_{g,m}=\tau_{g}+\Delta \tau_{g,m},$$

where $\tau_{g}$ denotes the immeasurable generator torque, $\Delta \tau_{g,m}$ denotes the measurement error coming from the actuator fault. Similar to the treatment of the pitch subsystem, the converter can be modeled by the following linear form:

(11) $$\begin{array}{cc} \; & {\dot{x}}_{g}=-\alpha_{gc}x_{g}+\alpha_{gc}\left(\tau_{g,r}+\Delta \tau_{g,m}\right),\\ & y_{g}=x_{g}. \end{array}$$

The adaptive observer for the faulty converter is thus designed as follows:

(12) $$\begin{array}{cc} \; & {\dot{\hat{x}}}_{g}=-\alpha_{gc}{\hat{x}}_{g}+\alpha_{gc}\tau_{g,r}+\alpha_{gc}{\hat{f}}_{g}-L_{g}\left({\hat{y}}_{g}-y_{g}\right),\\ & {\hat{y}}_{g}=x_{g}, \end{array}$$

where ${\dot{\hat{x}}}_{g}$ and ${\hat{y}}_{g}$ are the estimated values of ${\dot{x}}_{g}$ and ${\hat{y}}_{g}$, respectively. $L_{g}$ denotes the observer gain, and ${\hat{f}}_{g}$ is the fault value to be estimated.

### 3.3. FAFE Algorithm

The linear models Equation (7) of the Blade and Pitch and Equation (11) of the Generator and Converter subsystems with considered faults can be uniformly expressed as:

(13) $$\begin{array}{cc} \; & \dot{x}\left(t\right)=Ax\left(t\right)+Bu\left(t\right)+Ef\left(t\right),\\ & y(t)=Cx(t), \end{array}$$

where $x\left(t\right)\in R^{n}$, $u\left(t\right)\in R^{m}$, $y\left(t\right)\in R^{p}$, and $f\left(t\right)\in R^{r}$ represent the immeasurable state vector, the input vector, the output vector, and the fault vector, respectively. *E* is a constant real matrix. In particular, E=B for both the Blade and Pitch and the Generator and Converter subsystems, according to Equations (7) and (11). The matrices *A*, *B*, *C* should satisfy the conditions of controllability and observability, under the condition that the rank of the matrices $\begin{array}{cccc} {}[B & AB & \cdots & A^{n-1}B] \end{array}$ and ${\left[\begin{array}{cccc} C & CA & \cdots & CA^{n-1} \end{array}\right]}^{T}$ should be *n*, namely $r(\begin{array}{cccc} {}[B & AB & \cdots & A^{n-1}B] \end{array})=n$ and $r({\left[\begin{array}{cccc} C & CA & \cdots & CA^{n-1} \end{array}\right]}^{T})=n$.

The adaptive observer based on (13) is thus constructed:

(14) $$\begin{array}{cc} & \dot{\hat{x}}\left(t\right)=A\hat{x}\left(t\right)+Bu\left(t\right)+E\hat{f}\left(t\right)-L\left(\hat{y}\left(t\right)-y\left(t\right)\right),\\ \; & \hat{y}\left(t\right)=C\hat{x}\left(t\right), \end{array}$$

where $\hat{x}\left(t\right)\in R^{n}$, $\hat{f}\left(t\right)\in R^{r}$, and $\hat{y}\left(t\right)\in R^{p}$ separately represent the estimated value of x(t), u(t), and y(t) obtained by the observer. $L\in R^{n\times p}$ is the observer gain matrix.

Denote the error dynamics as:

(15) $${\dot{e}}_{x}\left(t\right)=\left(A-LC\right)e_{x}\left(t\right)+Ee_{f}\left(t\right),$$

(16) $$e_{y}\left(t\right)=Ce_{x}\left(t\right),$$

where $e_{x}\left(t\right)=\hat{x}\left(t\right)-x\left(t\right)$, $e_{y}\left(t\right)=\hat{y}\left(t\right)-y\left(t\right)$, and $e_{f}\left(t\right)=\hat{f}\left(t\right)-f\left(t\right)$ represent the error between the estimated and actual value of the state, output, and fault, respectively.

To estimate the fault value $\hat{f}\left(t\right)$ in the adaptive observer in Equation (14), the conventional fault estimation algorithm was proposed based on Theorem 1.

**Theorem** **1.** *If exists a symmetric positive definite matrix $Q\in R^{n\times n}$ and matrix $F\in R^{r\times p}$, the following conditions can be satisfied,*

(17) $$Q\left(A-LC\right)+{\left(A-LC\right)}^{T}Q<0,$$

(18) $$E^{T}Q=FC,$$

*then the conventional fault estimation algorithm*

(19) $$\hat{f}\left(t\right)=-\Gamma F\int_{t_{f}}^{t}e_{y}\left(t\right)dt,$$

*realizes $\lim_{t\to \infty }e_{x}\left(t\right)=0$ and $\lim_{t\to \infty }e_{f}\left(t\right)=0$ under input u(t), and the matrix $\Gamma \in R^{r\times r}$ represents the learning rate.*

The conventional algorithm only considers the constant fault, whose derivative equals 0, which is $\dot{f}\left(t\right)=0$. However, for the time-varying faults, $\dot{f}\left(t\right)\neq 0$, leading to the unsatisfied stability in the conventional algorithm. Besides, the equation of the conventional algorithm only contains the integral term, meaning that if the selected learning rate is too small, the speed of convergence would be slow; whereas if the learning rate is too large, it would lead to the problem of large overshoot. Therefore, to improve the speed and accuracy of fault estimation for both constant faults and time-varying faults, we introduce the FAFE algorithm, which is defined in Theorem 2.

**Theorem** **2.** *Given scalars σ>0, μ>0, if exist symmetric positive definite matrices $Q\in R^{n\times n}$, $H\in R^{r\times r}$, and matrices $F\in R^{r\times p}$, $M\in R^{n\times p}$, such that the following conditions hold*

(20) $$E^{T}Q=FC,$$

(21) $$\left[\begin{array}{cc} \Xi & -\frac{1}{\sigma }\left(A^{T}QE-C^{T}M^{T}E\right)\\ {}* & -2\frac{1}{\sigma }E^{T}QE+\frac{1}{\sigma \mu }H \end{array}\right]<0,$$

*where $\Xi =QA+A^{T}Q-MC-C^{T}M^{T}$, M=QL, and *∗* denotes the symmetric elements, then the FAFE algorithm*

(22) $$\hat{f}\left(t\right)=-\Gamma F\left(e_{y}\left(t\right)+\sigma \int_{t_{f}}^{t}e_{y}\left(t\right)dt\right)$$

*realizes $e_{x}\left(t\right)$ and $e_{f}\left(t\right)$ uniformly ultimately bounded.*

Theorem 2 is proved in [17]. For the given scalars σ and μ, based on the Equations (20) and (21) in Theorem 2, we can apply the LMI toolbox of Matlab to calculate the corresponding matrices *Q*, *H*, *F*, and *M*. Then the observer gain matrices $L_{p}$ in Equation (8) and $L_{p}$ in Equation (12) can be obtained by solving $L=Q^{-1}M$. The estimated fault value ${\hat{f}}_{p}$ and ${\hat{f}}_{g}$ in these adaptive observers can also be obtained by calculating the Equation (22).

## 4. FTC Scheme

Based on the estimated information obtained online by the adaptive observers designed in Section 3, we design the following fault tolerant controller:

(23) $$u\left(t\right)=-K\hat{x}\left(t\right)-\hat{f}\left(t\right),$$

where *K* denotes the gain matrix of the controller.

By substituting Equation (23) into Equation (13), the following equation yields:

(24) $$\begin{array}{cc} \dot{x}\left(t\right) & =Ax\left(t\right)+B(-K\hat{x}\left(t\right)-\hat{f}\left(t\right))+Bf\left(t\right)\\ & =\left(A-BK\right)x\left(t\right)-BKe_{x}\left(t\right)-Be_{f}\left(t\right)\\ & =\left(A-BK\right)x\left(t\right)+\left[\begin{array}{cc} -BK & -B \end{array}\right]\left[\begin{array}{c} e_{x}\left(t\right)\\ e_{f}\left(t\right) \end{array}\right] \end{array}$$

To prove the stability of the system, we introduce a lemma, which can be referred to [36].

**Lemma** **1.** *For a scalar μ>0, real vectors x and y, and a symmetric positive definite matrix P, the following inequality holds:*

(25) $$2x^{T}y\leq \frac{1}{\mu }x^{T}Px+\mu y^{T}P^{-1}y,$$

Denote the following Lyapunov function:

(26) $$\begin{array}{cc} V(t) & =\rho e_{x}^{T}\left(t\right)Qe_{x}\left(T\right)+\rho \frac{1}{\sigma }e_{f}^{T}\left(t\right)\Gamma^{-1}e_{f}\left(t\right)\\ & +x^{T}\left(t\right)Nx\left(t\right)-\rho \frac{\mu }{\sigma }f^{T}\left(t\right)\Gamma^{-1}H\Gamma^{-1}f\left(t\right), \end{array}$$

where ρ is a positive scalar, $N\in R^{n\times n}$ is a symmetric positive definite matrix, *Q*, *H*, and scalars σ, μ are chosen from Theorem 2. Then,

(27) $$\begin{array}{cc} \dot{V}\left(t\right) & =\rho {\dot{e}}_{x}^{T}\left(t\right)Qe_{x}\left(t\right)+\rho e_{x}^{T}\left(t\right)Q{\dot{e}}_{x}\left(t\right)+2\rho \frac{1}{\sigma }e_{f}^{T}\left(t\right)\Gamma^{-1}{\dot{e}}_{f}\left(t\right)\\ & +{\dot{x}}^{T}\left(t\right)Nx\left(t\right)+x^{T}\left(t\right)N\dot{x}\left(t\right)-\frac{\mu }{\sigma }{\dot{f}}^{T}\left(t\right)\Gamma^{-1}H\Gamma^{-1}f\left(t\right)\\ & -\rho \frac{\mu }{\sigma }f^{T}\left(t\right)\Gamma^{-1}H\Gamma^{-1}\dot{f}\left(t\right)\\ & =\rho e_{x}^{T}\left(t\right)\left(Q\left(A-LC\right)+{\left(A-LC\right)}^{T}Q\right)e_{x}\left(t\right)\\ & -2\rho \frac{1}{\sigma }e_{f}^{T}\left(t\right)E^{T}Q\left(A-LC\right)e_{x}\left(t\right)\\ & -2\rho \frac{1}{\sigma }e_{f}^{T}\left(t\right)E^{T}QEe_{f}\left(t\right)-2\rho \frac{1}{\sigma }e_{f}^{T}\left(t\right)\Gamma^{-1}\dot{f}\left(t\right)\\ & +x^{T}\left(t\right)\left(N\left(A-BK\right)+{\left(A-BK\right)}^{T}N\right)x\left(t\right)\\ & +2x^{T}\left(t\right)N\left[\begin{array}{cc} -BK & -B \end{array}\right]\left[\begin{array}{c} e_{x}\left(t\right)\\ e_{f}\left(t\right) \end{array}\right]\\ & -\rho \frac{\mu }{\sigma }\left({\dot{f}}^{T}\left(t\right)+f^{T}\left(t\right)\right)\Gamma^{-1}H\Gamma^{-1}\left(\dot{f}\left(t\right)+f\left(t\right)\right). \end{array}$$

According to Lemma1,

(28) $$\begin{array}{cc} -2\frac{1}{\sigma }e_{f}^{T}\left(t\right)\Gamma^{-1}\dot{f}\left(t\right)\leq & \frac{1}{\sigma \mu }e_{f}^{T}\left(t\right)He_{f}\left(t\right)\\ & +\frac{\mu }{\sigma }{\dot{f}}^{T}\left(t\right)\Gamma^{-1}H^{-1}\Gamma^{-1}\dot{f}\left(t\right). \end{array}$$

Let $\zeta \left(t\right)=\left[\begin{array}{c} e_{x}\left(t\right)\\ e_{f}\left(t\right) \end{array}\right]$, $X=N\left(A-BK\right)+{\left(A-BK\right)}^{T}N$,

$$Y=\left[\begin{array}{cc} Q\left(A-LC\right)+{\left(A-LC\right)}^{T}Q & -\frac{1}{\sigma }{\left(A-LC\right)}^{T}QE\\ {}* & -2\frac{1}{\sigma }E^{T}QE+\frac{1}{\sigma \mu }H \end{array}\right],$$

the derivative of V(t) is written as:

(29) $$\begin{array}{cc} \dot{V}\left(t\right)\leq & \rho \zeta^{T}\left(t\right)Y\zeta \left(t\right)-\rho \frac{\mu }{\sigma }f^{T}\left(t\right)\Gamma^{-1}H^{-1}\Gamma^{-1}f\left(t\right)\\ & +x^{T}\left(t\right)Xx\left(t\right)+2x^{T}\left(t\right)N\left[\begin{array}{cc} -BK & -B \end{array}\right]\left[\begin{array}{c} e_{x}\left(t\right)\\ e_{f}\left(t\right) \end{array}\right]. \end{array}$$

Denote

(30) $$\begin{array}{cc} \alpha & =\lambda_{min}\left(-Y\right),\\ \beta & =\lambda_{min}\left(-X\right),\\ \gamma & =\lambda_{min}\left(\Gamma^{-1}H^{-1}\Gamma^{-1}\right),\\ \xi & =\left|\right|\left[\begin{array}{cc} -NBK & -NB \end{array}\right]\left|\right|, \end{array}$$

thus Equation (29) is represented as

(31) $$\begin{array}{cc} \dot{V}\left(t\right) & \leq -{\rho \alpha ||\zeta \left(t\right)||}^{2}-\rho \frac{\mu }{\sigma }{\gamma ||f\left(t\right)||}^{2}+{\beta ||x\left(t\right)||}^{2}\\ & +2\xi ||x(t)||\cdot ||\zeta (t)||\\ & \leq -{\rho \alpha ||\zeta \left(t\right)||}^{2}+{\beta ||x\left(t\right)||}^{2}+2\xi ||x\left(t\right)||\cdot \left||\zeta \left(t\right)|\right|\\ & =-\rho \alpha \left[{\left||\zeta \left(t\right)|\right|}^{2}-\frac{2\xi }{\rho \alpha }\left||x\left(t\right)|\right|\cdot \left||\zeta \left(t\right)|\right|+\frac{\xi^{2}}{\rho^{2}\alpha^{2}}{\left||x\left(t\right)|\right|}^{2}\right]\\ & \leq -\frac{1}{\rho \alpha }\left[\rho \alpha \beta -\xi^{2}\right]{\left||x\left(t\right)|\right|}^{2}. \end{array}$$

Therefore, if $\rho >\frac{\xi^{2}}{\alpha \beta }$, $\dot{V}\left(t\right)<0$, the controller (23) guarantees the faulty system stable.

According to Equation (24), the faulty system can be stable by selecting the appropriate gain matrix *K* to design the state feedback fault tolerant controller. The pole placement method is utilized to calculate the gain matrix *K*, which place poles of the system at the previously determined locations. For the state feedback system, the poles are the roots of the characteristic function

(32) |sI−(A−BK)|=0.

The desired closed-loop pole locations are not unique, and the selection is arbitrary. However, the determined poles should be self-conjugated and located on the left-half side of the complex to stabilize the system and tolerate the faults. Similar strategies can also refer to [8,16]. For the blade and pitch system, we choose the pole as:

(33) $$P_{p}=\left[\begin{array}{cc} -7.775+7.9341i & -7.775-7.9341i \end{array}\right],$$

then the gain matrix is obtained:

(34) $$K_{p}=\left[\begin{array}{cc} -0.0040 & -0.0321 \end{array}\right].$$

For the generator and converter system, the pole is chosen as:

(35) $$P_{g}=-50.0050,$$

the corresponding gain matrix is thus calculated:

(36) $$K_{g}=\left[0.0001\right].$$

## 5. Simulation Results

The simulation results in this section illustrate the effectiveness of the proposed adaptive observer based AFTC scheme. The simulation was carried out within MATLAB/ SIMULINK in an Intel Core i5 CPU@ 2.3 GHz, Memory RAM 8.00 GB, MacOS 10 64-bit. The total operating time was set to 4400 s, and the sampling frequency was 100 Hz. To verify the effectiveness of the proposed scheme, we implemented the method in the reference [37] as the baseline.

### 5.1. Fault Estimation Comparison

**Figure 2:**
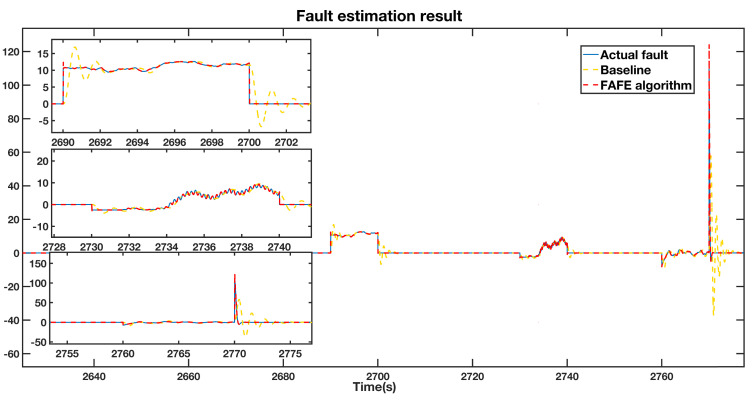
Fault estimation result adopting the FAFE algorithm and conventional algorithm.

We compare the fault estimation results of the FAFE algorithm and the baseline [37] concerning the measurement of pitch 2 by the first sensor $\beta_{2,m1}$. As denoted by the blue line in Figure 2, a fixed value fault of $\beta_{2,m1}=30^{\circ }$ from 2690 s to 2700 s, a gain factor fault of $\beta_{2,m1}^{\prime }=3.5\beta_{2,m1}$ from 2730 s to 2740 s, and an actuator fault of ζ=0.85, $\omega_{n}=2.03$ from 2760 s to 2770 s were injected to $\beta_{2,m1}$, respectively. We illustrate the detailed results in the left section of Figure 2. The fault estimation results of the FAFE algorithm and the baseline are compared, depicted by the red dotted line and yellow dotted line, respectively. According to Figure 2, both algorithms could successfully estimate the faults, whereas the FAFE algorithm estimates the actual fault values faster and has higher accuracy than the baseline. As can be seen from the three enlarged subfigures on the left side of Figure 2, when the fixed value and gain factor fault occur, the results of the baseline show oscillations of about 3 seconds before successfully tracking the fault values, while the estimated values of the FAFE algorithm coincide with the actual value during the whole period. Besides, it takes the baseline of more than 2 s to trace back $\beta_{2,m1}$ after returning to normal. However, the FAFE algorithm responds to the faults and fault free situation much faster and more precisely.

### 5.2. AFTC Simulations

To simulate the sensor faults, we injected two fixed value faults to $\beta_{1,m1}$ and $\beta_{3,m1}$ and a gain factor fault to $\beta_{2,m2}$, according to Table 2. In fact, the time periods of the faults are set randomly in the simulation, because the proposed scheme is independent of the time location of the faults, which provides good robustness toward the changed point of operation of the faults. More precisely, $\beta_{1,m1}$ is the measured pitch angle 1 value by the first sensor. It was set to a fixed value of $5^{\circ }$ from the 1000 s to 1100 s. $\beta_{3,m1}$ represents the measured pitch angle 3 value from the first sensor, inflicted by a fixed value of $6^{\circ }$ during the 2600 s to 2700 s. From the 2400 s to 2500 s, $\beta_{2,m2}$, the measured pitch angle 2 from the second sensor, experienced a gain of 1.2 times the normal value, resulting in $\beta_{2,m2}^{\prime }=1.2\beta_{2,m2}$. The actuator faults were injected into the two critical subsystems. For the blade and pitch subsystem, the parameters $\omega_{n}$ and ζ in Equation (4) were set to 11.11 and 0.7 in the normal operation condition. We changed their values for pitch 2 $\beta_{2}$ and pitch 3 $\beta_{3}$, respectively, in order to trigger dynamic changes in the pitch system. For the generator and converter subsystem, we added an offset to the generator torque $\tau_{g}$. After that, the proposed AFTC method was applied to the benchmark model.

#### 5.2.1. FTC Result of the Blade and Pitch System

**Figure 3:**
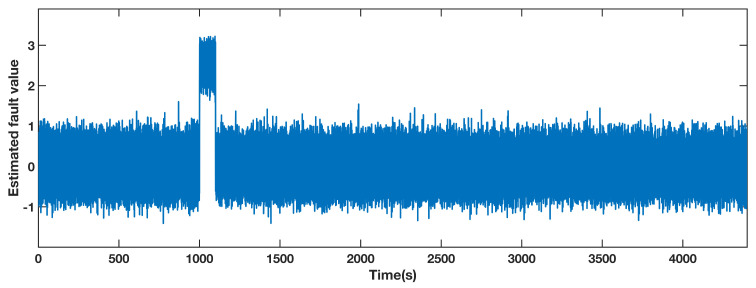
Estimated fault value from adaptive observer designed for the pitch angle 1.

The estimated fault value obtained by the adaptive observer designed for pitch 1 is shown in Figure 3. The measured value of the sensor contains both the actual value and the noise in the benchmark model. Due to the effect of the noise, the estimated fault value is within the range of [−1.5, 1.5], while it deviates from this range from 1000 s to 1100 s. Therefore, the fault can be located in pitch 1 according to the performance of the estimated fault.

**Figure 4:**
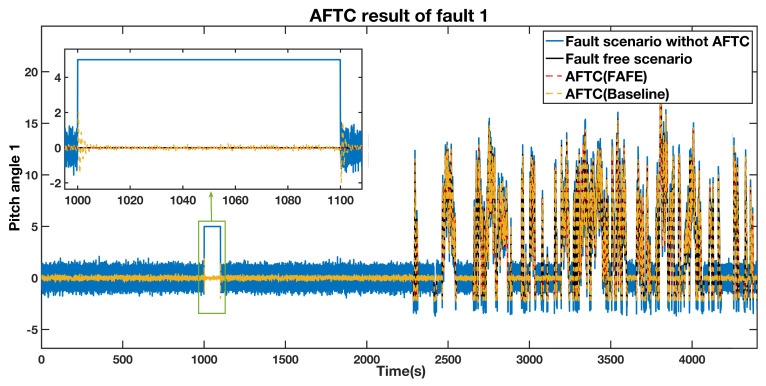
AFTC Results Comparison of the pitch angle 1 output.

The pitch angle 1 output in the injected fault and fault-free cases are illustrated in Figure 4, represented by the blue line and the black line separately. We adopted the proposed AFTC method to tolerate the sensor fault, and the result is represented by the red dashed line. This line approaches the black line during the faulty period, which means that the proposed AFTC scheme effectively recovers the faulty system to the nominal situation. Besides, we also designed the adaptive observer proposed in [37] to realize the AFTC, which is displayed by the yellow dashed line. It also approaches the black line from 1000 s to 1100 s. However, the red dashed line approaches the normal pitch angle value faster and closer than the yellow dashed line. Therefore, utilizing the FAFE algorithm has a better performance than the baseline to achieve the AFTC goal. The detailed comparison over the segment from 1000 s to 1100 s is also shown in the top left section of the figure.

**Figure 5:**
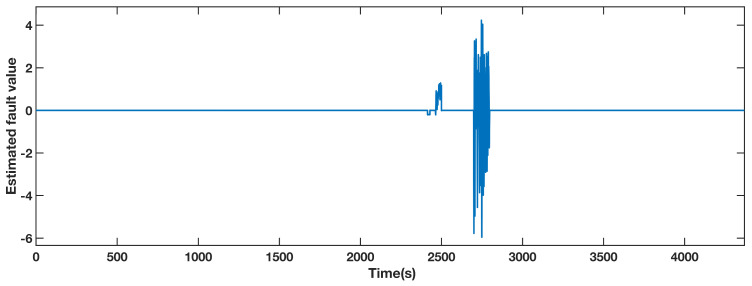
Estimated fault value from adaptive observer designed for the pitch angle 2.

**Figure 6:**
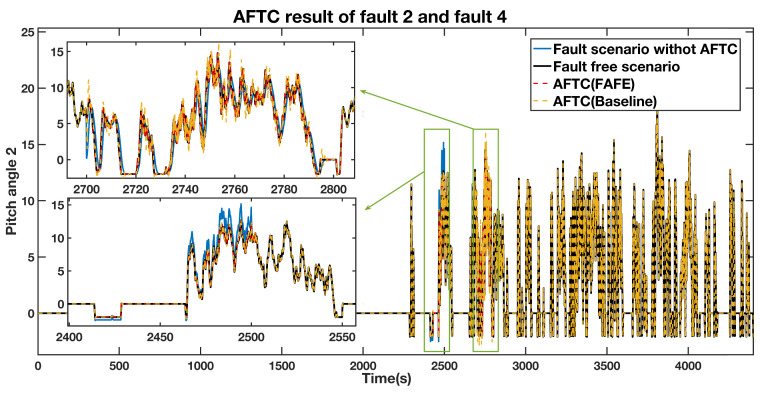
AFTC Results Comparison of the pitch angle 2 output.

A gain factor of 1.2 times the normal measured value of sensor fault during 2400 s to 2500 s and an actuator fault from 2700 s to 2800 s were injected to the pitch 2. The estimated fault value from the adaptive observer is displayed in Figure 5. The estimated fault obviously diverges from zero during the faulty periods, thus locating the fault. The pitch angle 2 AFTC outputs are compared in Figure 6. The details of the actuator fault and the gain factor fault are shown in the top left section and the bottom left section, respectively. The tendencies of the red and yellow dashed line, representing the fault scenario with AFTC by adopting the FAFE algorithm and the comparative reference [37], maintain normal. In the case of sensor factor fault from 2400 s to 2500 s, both the red line and the yellow line are close to the black line, representing the healthy operating output. Nevertheless, the FAFE algorithm shows better accuracy than the baseline. Therefore, the AFTC scheme can address the faults that occur on pitch 2, and adopting the FAFE algorithm also enhances the performance of this component.

**Figure 7:**
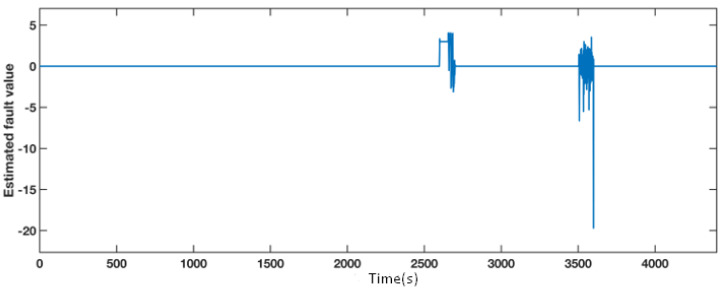
Estimated fault value from adaptive observer designed for the pitch angle 3.

**Figure 8:**
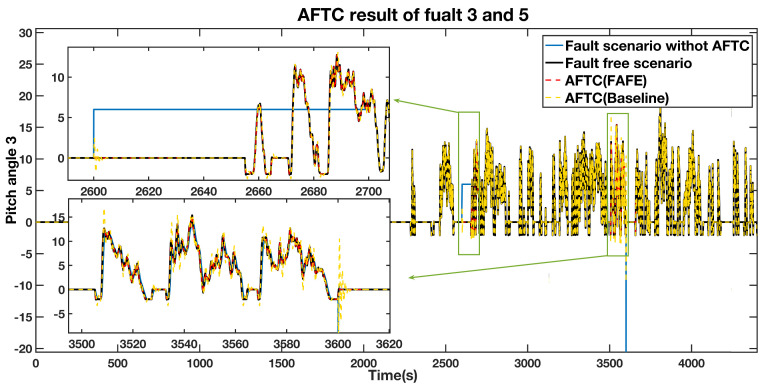
AFTC Results Comparison of the pitch angle 3 output.

A fixed value fault equals $6^{\circ }$ from 2600 s to 2700 s, and an actuator fault from 3500 s to 3600 s affect pitch 3. The simulation results are presented in Figure 7 and Figure 8. According to Figure 7, the blue line, which represents the estimated fault, significantly diverges from the normal operation during the faulty time. Therefore, we can deduce that the faults occur during these two periods. The four situations are also compared in pitch 3, as shown in Figure 8, and a detailed comparison of the sensor fault and the actuator fault are depicted in the top left and the bottom left section. During the faulty period, the AFTC scheme adopting the FAFE algorithm represented by the red dashed line and the baseline illustrated by the yellow dashed line recover to normal as the fault-free case denoted by the black line. Besides, the red line is closer to the black line than the yellow one. Therefore, the proposed method can achieve the AFTC requirement for pitch 3, and the fault tolerant result is effectively improved by applying the FAFE algorithm in the adaptive observer.

#### 5.2.2. AFTC Result of the Generator and Converter System

**Figure 9:**
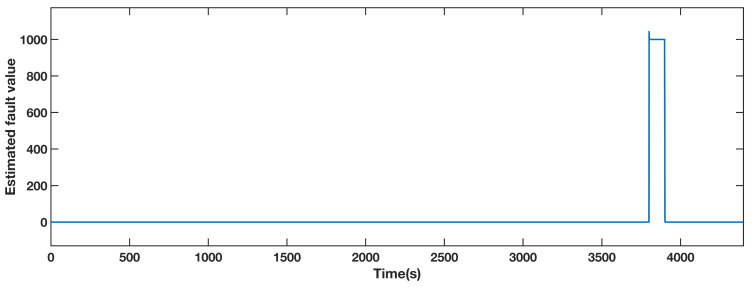
Estimated fault value from adaptive observer designed for the generator torque.

**Figure 10:**
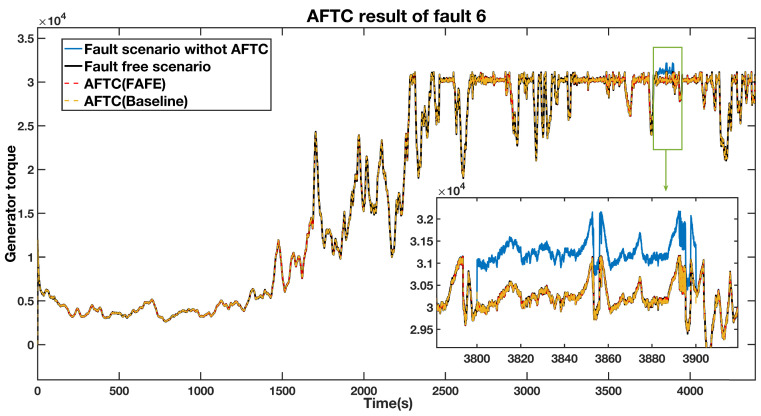
AFTC Results Comparison of the generator torque output.

An actuator fault from 3800 s to 3900 s causes a generator torque an offset of 1000 Nm. Figure 9 shows the estimated fault obtained by the FAFE algorithm. The blue line is the output value, and it significantly deviates from 0 between 3800 s to 3900 s, thus the fault can be located. Figure 10 illustrates the output of the generator torque under four conditions, and the bottom right section shows the details of the faulty period. Both the red and the yellow dashed lines, which represent the AFTC schemes adopting the FAFE and the baseline, recover to the normal operation state. Therefore, it can be deduced that the FTC method perfectly tolerates the actuator fault in this subsystem.

**Figure 11:**
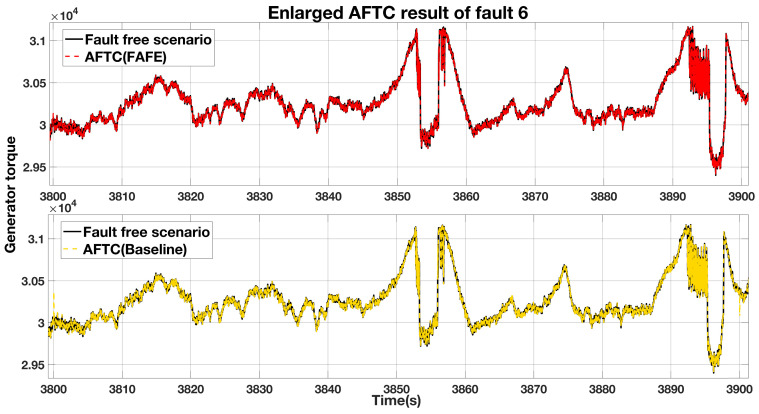
Enlarged comparison of the generator torque output.

To compare the FAFE algorithm and the baseline in this subsystem, we enlarge the faulty period in Figure 10 and re-contrast in Figure 11. The comparison between the fault-free case and the AFTC scheme with the FAFE algorithms is shown at the top of the figure, and the comparison with the baseline is displayed at the bottom. From Figure 11, we can see that both the AFTC schemes stabilize the faulty system and have similar performance. However, the FAFE algorithm performs better at the beginning and the end of the faulty period.

## 6. Conclusions

An adaptive observer based fault tolerant control method for the sensor and actuator faults on the blade and pitch and the generator and converter subsystems of wind turbines is proposed in this article. We have completed the following work: (1) According to the measurement values of sensors, the wind turbine subsystems are remodeled to tolerate the sensor and actuator faults. Based on the faulty subsystem models, corresponding adaptive observers are designed.( 2) The fast adaptive fault estimation algorithm(FAFE) is applied in the adaptive observers to estimate the fault values. The estimation performance of the FAFE algorithm is improved in comparison with the conventional fault estimation algorithm. (3) By applying the estimated state and the fault information from adaptive observers, a state feedback fault tolerant control scheme is presented to achieve the active fault tolerant control. The Lyapunov function verifies the system stability, and the controller gain is calculated through the pole placement method. The simulation results illustrate that the active fault tolerant control scheme presented in this paper maintains the stability of the wind turbine system with sensor and actuator faults. Furthermore, the system performance successfully recovers to normal operation during the faulty time.

In this study, we proposed the fault tolerant control scheme for the two critical components of wind turbines. However, the system faults which may occur in the drive train subsystem have not been solved yet. According to the noise and the disturbance, this subsystem model is nonlinear, different from the linear system we considered in this study. The fault tolerant method for the drive train subsystem is the main topic for our future work. Besides, this scheme is proposed based on the simulation, we will do more experiments according to data from the real wind farm.

## Figures and Tables

**Table 1:** Fault scenarios in the benchmark model.

Fault Scenario	Type	Faulty Component
Sensor fault	Fixed value	Blade and Pitch
Sensor fault	Gain factor	Blade and Pitch
Actuator fault	Change dynamics	Blade and Pitch
Actuator fault	Offset	Generator and Converter

**Table 2:** The setting values of considered fault scenarios.

Fault No.	Fault Scenario	Type	Fault Value	Time Period
1	Sensor fault	Fixed value	$\beta_{1,m1}=5^{\circ }$	1000–1100 s
2	Sensor fault	Gain factor	$\beta_{2,m2}^{\prime }=1.2\beta_{2,m2}$	2400–2500 s
3	Sensor fault	Fixed value	$\beta_{3,m1}=6^{\circ }$	2600–2700 s
4	Actuator fault	Change dynamics	$\zeta =0.85,\omega_{n}=2.03$	2700–2800 s
5	Actuator fault	Change dynamics	$\zeta =0.9,\omega_{n}=3.42$	3500–3600 s
6	Actuator fault	Offset	$\tau_{g}=\tau_{g}+1000$ Nm	3800–3900 s
